# Genetic variability for vigor and yield of robusta coffee (*Coffea canephora*) clones in Ghana

**DOI:** 10.1016/j.heliyon.2022.e10192

**Published:** 2022-08-11

**Authors:** Abraham Akpertey, Esther Anim-Kwapong, Paul Kwasi Krah Adu-Gyamfi, Atta Ofori

**Affiliations:** a Cocoa Research Institute of Ghana, P. O. Box 8, New Tafo-Akim, ER, Ghana; b Seed Production Division, Ghana Cocoa Board, P. O. Box 3197, Accra, Ghana

**Keywords:** Genetic correlation, Genetic parameters, Genetic variation, Germplasm, Heritability

## Abstract

Effectiveness of plant improvement programs, especially in perennial crops like coffee, may be improved by knowing the level of genetic variation which exist within a breeding population coupled with the knowledge of estimates of genetic parameters for key agronomic traits. Thus, this study was conducted to evaluate *C. canephora* clones for growth (stem diameter, number of laterals and span) and yield traits; estimate genetic parameters of these traits; and determine the phenotypic and genetic associations between these traits to guide future crop improvement efforts. The productivity of 56 coffee clones was assessed from 2012 to 2020 in a clonal experiment planted in a randomized complete block design with three replicates. Each plot consisted of eight plants spaced at 2 × 3 m at the Cocoa Research Institute of Ghana. There were significant (*p* < 0.001) differences among clones for all the traits assessed. Broad sense heritability was low-to-moderate for all the traits evaluated with the highest (0.34) observed for cumulative yield (CY). Cumulative yield was genetically correlated (*p* < 0.001) with span and number of laterals (NOL). For the growth traits, NOL was the most strongly associated with CY (*r*_*g*_ = 0.49, *p* < 0.001). The results revealed that selection based on early years’ yield (MY1) could be as effective as selection based on CY (*r*_*g*_ = 0.87, *p* < 0.001). Our findings indicate that there is significant genetic variation among the test clones for the parameters assessed and presents a good opportunity for future variety development.

## 1 Introduction

Coffee, a valuable stimulant crop belongs to the genus *Coffea*, sub-genus *Coffea*, and the family Rubiaceae. It grows typically in tropical and subtropical countries worldwide (DaMatta, 2004; Ferreiraa et al., 2019). *C. arabica* (typically referred to as Arabica) and *C. canephora* Pierre (typically referred to as Robusta) are the two widely cultivated species of the 126 species classified under the genera *Coffea* (Davis et al., 2019). *C. arabica* is an allotetraploid (2n = 44) which is predominantly self-pollinating whereas *C. canephora* is a diploid species (2n = 22) which is mainly out-crossing. Robusta coffee in comparison with Arabica coffee is known for its better productivity and higher caffeine content, enhanced growth at lower altitude, and better tolerance to biotic and abiotic stresses (DaMatta et al., 2007). Robusta coffee which has a much wider distribution than Arabica coffee (Hendre and Aggarwal, 2014) is the main coffee type grown in Ghana, mainly due to ecological adaptation reasons.

In Ghana both hybrid and clone varieties of Robusta coffee are deployed to farmers with the aim of improving and increasing total national production. Generally, similar to other tree crops, low coffee yields have been reported in Ghana and elsewhere in West Africa. In the country, on-farm yields of coffee in the range of 0.1–0.2 t ha^−1^ (Anonymous, 1996) have been reported. This is contrary to the relatively high yields (>2 t ha^−1^) recorded in on-station trials (Akpertey et al., 2018; Anim-Kwapong et al., 2011). It has been posited that the use of unimproved planting materials is one of the main causes of poor coffee yields in Ghana. National coffee production has been around some 6000 tons on average in Ghana. In recent times however, a national coffee production of 12,650 tons in 2015 was attributed in part to the deployment of improved varieties during a 4-year coffee Rehabilitation program which was initiated in 2011 in the country (ICO, 2018). This underscores the importance of high-yielding varieties in improving production per unit land area and total national productivity. The need to develop high-yielding varieties with improved qualities can therefore, not be overemphasized.

Breeding programs of *C. canephora* explore novel strategies to assess more genotypes within a short time period, thus increasing the potential to exploit genetic variability for crop improvement (Dubberstein et al., 2017; Rodrigues et al., 2016; Rocha et al., 2015). Evaluation of clones in extensive trials is one approach employed by many breeding programs to assess genetic variability and select high-yielding clones with desired characteristics (Souza et al., 2017; Santos et al., 2017). Improved clonal varieties or accessions play a significant role in any crop improvement program, particularly serving as parents in hybrid variety development or planted directly as clonal varieties. The lack of adequate agronomic and genetic parameter information on such clones may therefore, hamper their selection and incorporation into hybrid and or clone breeding programs. Additionally, after extensive field evaluations, the best clones based on important target traits can be recommended as varieties for farmers in target regions. Therefore, it is of major importance during the process of selection, to quantify the level of genetic and environmental variances, in order to better understand gene action underlying heritability of important target traits (Vencovsky and Barriga, 1992). This will go a long way to inform the best breeding strategies to adopt for the speedy improvement of particular traits within a population. Additionally, Gichimu and Omondi (2010) posited that the continuous development of new coffee varieties requires that the level and sources of genetic variation within and between new and existing coffee genotypes be determined. Such information will help a breeder to easily quantify the progress being made in the development of better varieties for farmers and modify breeding strategies where necessary to exploit to the maximum, the existing genetic diversity in coffee improvement.

In plant breeding, knowing the properties of target traits at the genetic level is a pre-requisite in establishing a proper crop improvement program. Particularly, there is a requirement to estimate the heritability and inter-relationships of important agronomic parameters. Because most quantitative traits are complex in nature and are greatly influenced by the environment, selection of superior progenies is onerous (Cruz et al., 2004). Nonetheless, knowledge and understanding of the genetic basis for target traits would help surmount some of the challenges usually associated with selection for quantitative traits that are complex in nature; and facilitate success of a breeding program (Mistro et al., 2004). Oliveira et al. (2010) suggested that to make significant gains in variety development, it is important to estimate and define both the genetic variability in the species as well as the relationships among important agronomic traits. Estimates of genetic parameters are important so as to enhance the selection of better genotypes and effectively assess and explore other breeding strategies (Hardner et al., 2001). Reuben et al. (2003) posited that, to develop improved varieties, it is important for coffee breeders to estimate genetic parameters and determine relationships among target traits as this will enhance the breeding process through indirect selection. Also, Oliveira et al. (2008) suggested that estimations of genetic parameters such as genotypic variance and heritability allows for better understanding of the gene action underlying how traits are inherited and facilitates better evaluation of expected improvement with selection in addition to defining the appropriate selection strategy to be employed.

Associations between traits have received considerable attention from plant breeders because of the importance and economics of reducing breeding cycles as well as increasing selection gain of important traits per unit time (Namkoong and Kang, 1990). From a breeding perspective, correlation analysis serves three important roles. Firstly, it can help to identify associations among target traits and how selection among traits impact each other (Hardner et al., 2001). Secondly, it helps to identify new parental combinations for variety development and finally, help to detect trait measurement redundancy (Yan, 2008). Selection for traits that are strongly associated with other traits could result in savings in cost of redundant data collection. Although, in facilitating breeding of Robusta coffee, several studies have investigated the significance of genetic parameters and associations among target traits, there exist some discrepancies in the magnitude of genetic parameters of important agronomic traits to enhance crop improvement efforts (Walyaro and Van der Vossen, 1979; Leroy et al., 1994; Abdi, 2009; Atinafu et al., 2017; de Oliveira et al., 2018; Akpertey et al., 2018). It is worth noting however, that findings of some studies have established significant differences among coffee genotypes as well as significant associations among important growth and yield traits have been detected (Akpertey et al., 2018, 2020; Anim-Kwapong et al., 2011; Cilas et al., 2006; Montagnon et al., 2003; Leroy et al., 1997). The objectives of the study were to evaluate Robusta coffee clones for growth and yield traits; estimate genetic parameters of these traits; and determine the phenotypic and genotypic correlations among the growth and yield traits to direct future Robusta coffee breeding efforts.

## 2 Materials and methods

### 2.1 Plant material

Fifty-six (56) clones that were derived through ortet selection based on yield of earlier hybrid experiments at the Cocoa Research Institute of Ghana (CRIG) were used in the study. The ortet selection process followed a procedure where the best performing plants in terms of growth and yield from the best families of previous hybrid trials were selected and cuttings were obtained from them to produce the clones (obtained through rooting of single-node cuttings in propagators) evaluated in this study. The rooting media in the propagators was sand and rice husk mixed in a 1:1 ratio. After six months of growth in the poly bags at the nursery, the rooted seedlings were field transplanted.

### 2.2 Evaluation of clone performance in the field

The field experiment was carried out at CRIG which is located at New Tafo-Akim (latitude 06° 13'N, longitude 0° 22'W) in the Eastern Region of Ghana. The soil at Tafo is sandy loam, brown to yellowish red, well drained, and developed in situ from weathered materials of hornblende granodiorite and classified as Haplic Luvisol (Adu & Asiamah, 1992). The information on weather parameters including rainfall, temperature and relative humidity during the period of the study was obtained from the meteorological station of CRIG (Table 1). The 56 clones were field established following a randomized complete block design with three replicates in June 2012. Each plot consisted of 8 plants at a spacing of 2 × 3 m. *Gliricidia sepium* was planted between rows at a spacing of 4 × 6 m to provide shade which was managed by pruning yearly. Following the 2 × 3 m planting distance used in the experiment, the total number of trees per hectare was 1667. During the field evaluation, each test plant grew on a maximum of two stems. Field assessment of the test plants were conducted following standard procedures for Robusta coffee cultivation in Ghana (Oppong et al., 2016). The test plants were not fertilized during the study period. Three growth traits including plant stem diameter (mm), span (m) and number of laterals were assessed over a 4-year period (2012–2015) whereas yield (t ha^−1^) was measured over a 6-year period (2015–2020).

**Table 1:** Rainfall, temperature and relative humidity during the period of the study between 2012 and 2020.

Year	Mean temperature °C				
	Total rainfall (mm)	Number of days	Minimum (0900h)	Maximum (1500h)	Mean relative humidity at 1500 h (%)
2012	1317.1	128	20.9	31.6	65.9
2013	1516.2	123	19.4	31.8	63.0
2014	1748.8	132	18.5	31.6	68.0
2015	1088.1	106	18.8	32.3	60.9
2016	1642.9	111	20.4	32.6	62.9
2017	1650.9	111	22.7	32.2	66.5
2018	1423.7	108	23.1	32.1	64.7
2019	1478.0	97	23.0	32.3	66.2
2020	1397.7	88	19.6	32.4	61.8
Mean	1473.7	111.6	20.7	32.1	64.4
SEM	66.6	4.8	0.6	0.1	0.8

SEM = Standard error of mean.

### 2.3 Data collection

Using electronic calipers, stem diameter of each test plant at 10 cm above the soil surface was measured. Span (m), a measure of canopy width was taken where tree canopy was the widest. For each tree, the number of laterals was counted at each data collection time. In instances where a test plant had more than one stem, stem diameter was estimated following Stewart and Salazar (1992). Similarly, where the test plant had more than one stem, span measurement was taken on only the largest stem. Stem diameter, number of laterals and span were measured once every year from 2012 to 2015. Cherry weight (fresh weight) was recorded for each test plant for six productive years from 2015 to 2020 and conversion of cherry weight to clean coffee yield (dry bean weight) was done using an outturn (dry bean weight/fresh weight) conversion factor of 0.22 (Wellman, 1961; Coste, 1992).

### 2.4 Data analysis

An analysis of variance (ANOVA) based on a linear model as in Eq. (1) was carried out for each trait:

(1) Y_ij_ = μ + g_i_ + b_j_ + e_ij_

where Y_ij_ is the phenotypic measurement of the i^th^ clone in the j^th^ block for trait Y, μ is the population mean, g_i_ is the effect of the i^th^ clone, b_j_ is the effect of the j^th^ block and e_ij_ is the error term. Additionally, in accounting for uncontrolled heterogeneity due to random factors, a linear mixed model analysis procedure was explored where clone and block were considered as a random and fixed factors, respectively. As a measure of plant vigor, data on stem diameter used in the analysis was estimated as the change between final and initial trait measurements in 2015 and 2012, respectively. Number of laterals and span data used in the analyses were restricted to the final vegetative trait measurements in 2015 which was the start of the reproductive growth phase of the crop. The ANOVA following tests for normality (based on the plot of residuals) was carried out with the ADEL-R statistical tool (Pacheco et al., 2016).

Genetic correlations (*r*_*g*_) between traits were estimated following Cooper et al. (1996) as in Eq. (2):

(2) $$\;r_{g}\text{ ​}=\frac{AM\;\left[\sigma g\left(jj^{\prime }\right)\right]}{AM\;\left[\sigma^{2}g\;\left(j\right)\;\sigma^{2}g\;\left(j^{\prime }\right)\right]\;}$$

where $AM\;\left[\sigma g\left(jj^{\prime }\right)\right]$ is the arithmetic mean of all pairwise genotypic covariances between trait j and $j^{\prime }$ and $AM\;\left[\sigma^{2}g\;\left(j\right)\;\sigma^{2}g\;\left(j^{\prime }\right)\right]$ is the arithmetic average of all pairwise geometric means among the genotypic variance components of the traits. Similarly, Pearson’s phenotypic correlation coefficients were estimated among all evaluated traits. To assess the possibility of selection for high-yielding genotypes based on cumulative yield (CY) early in a breeding program, genotypes were grouped according to their first three-year (2015–2017) yields (MY1) and last three-year (2018–2020) yields (MY2) to establish a relationship among selection based on early, late and cumulative yields.

Variance components were assessed and used to estimate broad sense heritability (H^2^) for each trait on an error plot variance (entry-mean) basis following Knapp et al. (1985) as in Eq. (3):

(3) $${\text{H}}^{2}\text{ ​}=\;\frac{\sigma ²g\;}{\left(\sigma^{2}g+\;\sigma^{2}e/nr\right)\;}$$

where *σ*^*2*^_*g*_ and *σ*^*2*^_*e*_ are genotype and error variance components, respectively, and *nr* is the number of replications. Only broad sense heritability estimations were possible due to the clonal nature of the genotypes assessed in the trial.

Following Burton (1952), GCV and PCV were estimated as in Eq. (4) and Eq. (5), respectively:

(4) $$\;\text{GCV}=\frac{\surd \sigma ²g}{\text{M}}\times 100$$

and

(5) $$\text{PCV ​}=\;\frac{\surd \sigma ²p}{\text{M}}\times 100$$

where GCV is the genotypic coefficient of variation, PCV is the phenotypic coefficient of variation, M is the trait mean and *σ*^*2*^_*g*_ and *σ*^*2*^_*p*_ are the genotypic and phenotypic variance, respectively.

Genetic advance as a percentage of the mean (GAM) for each trait was estimated following Falconer and Mackay (1996) as in Eq. (6):

(6) $$\text{GAM ​}=\frac{({\text{k ​x ​ ​H}}^{2}\text{x ​}\sqrt{\sigma^{2}p)}}{\text{M}}\times 100$$

where, k = the selection differential at 5% selection intensity (k = 2.063); H^2^ = broad sense heritability; *σ*^*2*^_*p*_ = phenotypic variance and M = trait mean.

Heritability and correlation analysis were carried out using the METAR-R statistical package (Alvarado et al., 2016).

## 3 Results

### 3.1 Weather characteristics of the experimental site

A mean rainfall of 1473.7 mm per annum was received during the study period. The least and highest amount of rainfall were received in the years 2015 (1088.1 mm) and 2014 (1748.8 mm), respectively (Table 1). The accompanying number of rainy days were lowest in year 2020 (88) and highest in year 2014 (132). Generally, in all years, there was a marked 4-month dry period that occurred between the months of November and February (data not presented). The mean annual temperature noted during the study period ranged from 18.5 °C in year 2014 to 32.6 °C in year 2016. Similarly, relative humidity recorded during the study period ranged from 60.9 % in year 2015 to 68.0 % in 2014 (Table 1).

### 3.2 Variation and agronomic performance of evaluated robusta coffee clones

Analysis of variance (ANOVA) revealed a highly significant (*p* < 0.001) difference among the Robusta coffee clones for all the assessed traits (Table 2). Similarly, with the exception of span, 2017 yield and 2019 yield, there was a significant (*p* < 0.01) block effect for all traits (Table 2). Also, there was a significant (*p* < 0.01) effect of clone, year and clone × year interaction for all traits when a combined ANOVA was conducted (data not presented). The linear mixed model analysis where clones were considered as random factors also revealed significant differences among the clones for all the traits measured (Table S1). Generally, for all traits, it was observed that the agronomic performance of the clones for the most part were slightly over estimated in the analysis of variance where the clones were considered fixed compared to the linear mixed model analysis approach (Table S1). The opposite was however, observed for some clones. Regardless of the plasticity in trait performance estimation between the two analyses approaches (simple ANOVA vs a linear mixed model approach), the rankings of clones for each trait did not change. A clear example were clones H498 and E139 which were the least and highest yielding clones, respectively in terms of cumulative yield for both models of analysis. Given the similarity between the two analysis approaches, the proceeding report of trait performance of the clones was based on the simple ANOVA output.

**Table 2:** Mean squares of growth and yield traits from ANOVA of 56 Robusta coffee clones assessed.

Source of Variation	df	Growth traits^a^			Yield (t ha^−1^)^b^								
		SDI (mm)	Span (m)	NOL	2015	2016	2017	2018	2019	2020	MY1	MY2	CY
Block	2	1661.38∗∗∗	0.04ns	5324.14∗∗∗	17.08∗∗∗	1.69∗∗∗	0.95ns	15.93∗∗∗	5.49ns	5.03∗∗	3.66∗∗	10.49∗∗∗	75.40∗∗
Clone	55	241.23∗∗∗	0.47∗∗∗	1863.25∗∗∗	4.07∗∗∗	0.99∗∗∗	6.09∗∗∗	3.34∗∗∗	7.53∗∗∗	1.27∗∗∗	2.97∗∗∗	3.02∗∗∗	82.77∗∗∗
Residual	1217	121.71	0.14	449.79	1.08	0.24	2.03	1.83	1.94	0.33	0.90	0.99	16.87

∗∗∗, ∗∗, and ns denote significance at 0.001, 0.01, 0.05, and not significant, respectively.

a SDI = Stem diameter increment from 2012 to 2015; NOL = number of laterals.

b MY1 = First three years mean yield (2015–2017); MY2 = last three years mean yield (2018–2020); CY = cumulative yield from 2015 to 2020.

Stem diameter increments (SDI) ranged from 27.6 mm (H317) to 44.4 mm (E139 × C134) among the 56 clones evaluated, with the standard clones (E138 and E139) recording SDI of 33.1 mm and 37.2 mm, respectively (Table 3). Distribution of SDI showed that only one clone (E139 × C134) was significantly (*p* < 0.05) better than the best standard clone (E139). On the other hand, five clones (H408, H404, H205, H222 and E139 × C134) were found to be significantly better in terms of SDI than the second standard clone (E138) over a 4-year (2012–2015) vegetative growth period (Table 3). Span varied from 1.4 m in clone H459 to 2.2 m in a standard clone, E139. The average span for all the 56 clones evaluated in this study was 1.9 m. On average, the clones with the least (33.5) and most (93.8) number of laterals were H459 and a standard clone E139, respectively. The standard clone E138 which had on average 42.9 laterals was not significantly different (*p* > 0.05) from the clone (H459) which had the least number of laterals (Table 3).

**Table 3:** Mean growth and yield trait measurements of 56 Robusta coffee clones assessed.

Clone^a^	Growth^b^			Yield (t ha^−1^)^c^								
	SDI (mm)	Span (m)	NOL	2015	2016	2017	2018	2019	2020	MY1	MY2	CY
A129	38.7	1.9	61.3	1.3	0.6	3.0	2.7	3.5	0.8	1.8	2.6	10.9
E138	33.1	1.6	42.9	0.7	0.4	1.8	2.0	2.0	0.5	1.0	1.9	5.6
E138 x C193	39.5	2.0	57.8	0.9	0.2	2.3	2.1	2.1	0.4	1.3	1.9	7.2
E139	37.2	2.2	93.8	3.4	0.9	4.4	2.9	4.4	0.6	3.1	3.0	15.5
E139 x C134	44.4	1.8	77.5	2.0	0.5	2.6	1.6	1.8	0.6	1.9	1.6	7.7
E139 x C134a	36.7	1.7	54.5	1.5	0.4	2.3	1.4	2.6	0.8	1.4	1.7	6.8
E139a	37.2	1.8	73.4	1.3	0.3	2.9	2.1	2.1	0.6	1.8	1.8	8.0
H1001	37.4	2.0	73.7	1.5	0.4	2.2	2.2	2.5	0.7	1.4	2.1	8.6
H1069	37.8	2.0	68.8	1.2	0.2	2.0	1.7	2.0	0.3	1.3	1.6	6.2
H1070	39.6	1.9	67.6	1.2	0.1	2.0	1.2	1.2	0.6	1.4	1.1	5.4
H116	37.1	1.8	70.5	1.0	0.2	2.3	1.8	1.5	0.5	1.6	1.5	6.2
H137	37.0	1.7	59.5	1.1	0.2	1.6	2.3	0.9	0.7	1.3	1.5	5.4
H204	38.2	2.0	69.7	0.9	0.1	1.9	1.8	1.5	0.5	1.3	1.5	5.7
H205	41.0	2.0	68.0	1.1	0.1	1.5	1.1	1.2	0.2	1.2	1.0	4.4
H207	36.5	2.0	68.1	1.4	0.3	2.4	2.1	2.7	1.1	1.7	2.3	8.9
H210	34.2	1.8	68.6	1.2	0.2	2.2	1.2	2.1	0.3	1.5	1.4	6.1
H222	41.7	2.1	76.7	1.5	0.1	2.8	2.2	2.3	1.0	1.9	1.9	9.0
H242	33.7	2.0	70.6	1.4	0.2	3.0	2.0	2.3	0.6	2.0	1.9	8.8
H246	36.1	1.8	69.0	1.0	0.4	2.0	1.9	2.1	0.7	1.3	1.8	6.9
H249	36.2	1.9	59.1	0.5	0.0	1.2	1.2	0.9	0.8	0.8	1.0	3.5
H250	39.2	2.1	75.6	0.9	0.3	2.1	2.2	1.8	0.4	1.4	1.8	6.8
H253	35.2	1.9	67.4	1.2	0.2	1.8	1.4	1.7	0.9	1.4	1.5	5.7
H317	27.6	1.6	61.5	0.9	0.3	1.8	1.4	1.9	0.6	1.1	1.2	5.1
H321	33.3	1.8	62.1	1.2	0.2	2.3	1.5	1.3	0.3	1.4	1.1	5.8
H324	37.9	1.9	62.8	0.8	0.3	1.4	1.4	1.2	0.4	1.1	1.2	4.5
H365	36.9	1.9	74.0	1.3	0.4	2.3	2.0	1.8	0.9	1.6	1.7	7.1
H388	32.1	1.7	59.9	1.0	0.0	1.7	1.8	1.5	0.2	1.3	1.4	5.3
H395	36.7	2.0	91.9	1.6	0.3	2.4	1.7	1.6	0.5	1.9	1.6	7.2
H404	40.4	1.7	69.0	1.2	0.7	2.3	0.9	1.4	0.3	1.6	1.0	5.4
H408	40.3	1.9	71.8	1.4	0.2	1.6	1.3	1.2	0.4	1.3	1.2	5.4
H429	36.5	1.9	66.4	1.1	0.3	2.2	2.3	2.0	0.4	1.3	1.9	6.9
H453	37.3	2.1	71.4	1.9	0.2	2.4	1.7	2.3	0.4	1.8	1.6	7.9
H459	28.0	1.4	33.5	0.4	0.5	1.3	1.5	1.3	0.2	0.9	1.3	4.2
H480	35.7	2.1	64.7	1.1	0.7	2.2	1.8	1.9	0.5	1.4	1.5	7.4
H497	31.4	1.8	57.5	1.2	0.1	2.4	1.8	1.9	0.3	1.5	1.4	7.2
H498	28.3	1.6	50.5	0.4	0.4	1.0	0.9	1.7	0.3	0.6	1.3	2.5
H502	37.3	2.0	75.6	1.1	0.2	1.8	1.5	1.7	0.2	1.3	1.4	5.5
H543	37.7	1.9	69.2	1.3	0.4	2.6	1.9	2.0	0.5	1.8	1.7	7.9
H55	35.6	1.9	68.3	1.4	0.2	2.0	1.8	1.6	0.4	1.5	1.5	6.4
H556	33.6	1.8	64.3	1.6	0.1	1.8	2.2	1.9	0.8	1.5	1.9	7.4
H574	39.5	2.0	68.8	1.0	0.9	2.1	1.5	1.7	0.4	1.4	1.4	6.3
H602	35.8	1.8	63.4	0.7	0.6	1.4	1.8	1.3	0.6	1.0	1.4	5.3
H622	33.2	2.0	70.5	0.9	0.1	2.1	1.5	1.2	1.0	1.4	1.3	5.6
H641	30.7	1.9	56.4	1.0	0.1	1.8	2.0	1.5	0.6	1.2	1.6	5.6
H643	33.0	2.0	56.3	1.2	0.1	2.3	1.8	1.7	0.8	1.5	1.6	7.0
H674	33.5	1.8	70.1	1.0	0.1	1.8	1.3	1.2	0.3	1.3	1.2	5.3
H707	34.4	1.9	61.7	1.6	0.2	2.0	1.4	1.8	0.3	1.5	1.5	6.2
H727	31.6	1.9	60.7	1.0	0.0	1.8	1.7	1.6	0.2	1.1	1.3	4.3
H729	34.2	1.8	63.0	1.4	0.1	2.4	1.4	2.1	0.3	1.7	1.5	6.9
H760	32.6	1.9	69.4	1.9	0.4	2.9	2.4	2.4	0.6	2.1	2.0	9.9
H764	38.0	1.9	74.0	1.2	0.2	1.8	1.7	1.8	0.4	1.4	1.6	6.3
H765	34.2	2.0	64.0	1.2	0.1	1.6	1.6	1.1	0.2	1.2	1.1	5.5
H885	31.1	1.7	60.9	1.1	0.2	1.7	1.8	1.6	0.2	1.2	1.5	6.0
H898	36.4	2.0	68.9	1.1	0.2	2.4	1.9	1.7	0.6	1.5	1.7	6.9
H907	38.1	2.1	64.6	2.0	0.6	2.3	1.8	1.4	0.6	2.0	1.4	6.9
H957	35.8	1.8	67.5	1.4	0.3	2.1	2.1	1.8	0.4	1.5	1.6	6.7
Mean	35.8	1.9	66.2	1.2	0.3	2.1	1.8	1.8	0.5	1.5	1.6	6.6
Lsd (*p* < 0.05)	6.7	0.2	13.2	0.7	0.6	0.9	0.9	0.9	0.3	0.6	0.6	2.5

a E138 and E139 were standard clones included in the study.

b SDI = Stem diameter increment from 2012 to 2015; NOL = number of laterals.

c MY1 = First three years mean yield (2015–2017); MY2 = last three years mean yield (2018–2020) CY = cumulative yield from 2015 to 2020.

On average, annual yields were generally lower in early productive years of the crop compared with productivity in later years. Average yield in 2015 varied from 0.4 t ha^−1^ in clones (H498 and H459) to 3.4 t ha^−1^ in the standard clone, E139 with a mean of 1.2 t ha^−1^ (Table 3). Yield in 2016 was generally low among the 56 Robusta coffee clones assessed with a mean yield of 0.3 t ha^−1^ recorded. However, yields increased in 2017 compared with the preceding year, ranging between 1.0 t ha^−1^ in clone H498 to 4.4 t ha^−1^ in the standard clone, E139. A similar yield trend was observed in 2018 where yield varied between 0.9 t ha^−1^ in clones (H498 and H404) and 2.9 t ha^−1^ in the standard clone, E139 (Table 3). The 2018 mean yield of all the clones assessed in the study was 1.8 t ha^−1^. Similar to yield trends in 2018, the mean yield of all the 56 clones evaluated was 1.8 t ha^−1^ in 2019. Yield in 2020 was generally low compared to the preceding year with yields ranging from 0.2 t ha^−1^ to 1.1 t ha^−1^ and an average of 0.5 t ha^−1^ for all the tested clones (Table 3). The first 3-year mean yield (MY1) among the evaluated clones varied from 0.6 t ha^−1^ in clone H498 to 3.1 t ha^−1^ in the standard clone, E139 with an average of 1.5 t ha^−1^. The last 3-year average yield (MY2) followed a similar trend varying from 1.0 t ha^−1^ in clones (H404, H249 and H205) to 3.0 t ha^−1^ in the standard clone, E139. Cumulative yield for all the clones over the 6-year period (2015–2020) ranged from a low of 2.5 t ha^−1^ in clone H498 to a high of 15.5 t ha^−1^ in one of the standard clones, E139 (Table 3). The cumulative yield distribution showed that the standard clone E139 was the highest yielding clone during the 6-year yield recording period of the study. This standard clone was generally consistent in its annual yields where it had high yields in high or low-yielding years. However, six clones (H1001, H242, H207, H222, H760 and A129) were significantly better than the second standard clone, E138 for cumulative yield. Also, approximately 46.4 % of the clones assessed had cumulative yields greater than the mean cumulative yield of 6.6 t ha^−1^ (Table 3).

### 3.3 Genetic variability and genetic parameter estimates

The error variance were larger than their respective genotypic variance, resulting in a more than 1 environmental variance to genetic variance ratio for all the traits assessed (Table 4). For the growth traits, the effect of the environment was more prominent on stem diameter increments (SDI) with a relatively high environmental variance to genetic variance ratio of 23.4. Similarly, for the yield traits, the highest environmental variance to genetic variance ratio was observed for 2020 yield. Number of laterals (6.8) and cumulative yield (CY) (5.7) were the growth and yield traits, respectively that were least influenced by the environment adjudged by the relatively low environmental variance to genetic variance ratio (Table 4).

**Table 4:** Genetic parameter estimates of growth and yield traits of 56 Robusta coffee clones assessed.

Trait^a^	Genetic parameter^b^						
	δ^2^_g_	δ^2^_e_	H^2^	GCV	PCV	δ^2^_e_/δ^2^_g_	GAM
SDI (mm)	5.20	121.78	0.11	6.36	18.89	23.43	4.42
Span (m)	0.02	0.14	0.24	6.48	13.20	9.45	6.56
NOL	65.77	450.00	0.30	12.24	22.18	6.84	13.95
Yield-15 (t ha^−1^)	0.15	1.08	0.29	31.12	57.78	7.34	34.57
Yield-16 (t ha^−1^)	0.02	0.23	0.17	43.75	105.22	14.35	37.53
Yield-17 (t ha^−1^)	0.19	2.03	0.22	20.80	44.21	10.56	20.19
Yield-18 (t ha^−1^)	0.07	1.83	0.11	15.36	46.96	25.04	10.37
Yield-19 (t ha^−1^)	0.27	1.94	0.29	28.29	52.46	7.32	31.47
Yield-20 (t ha^−1^)	0.01	0.33	0.06	16.65	65.58	43.51	8.73
MY1 (t ha^−1^)	0.09	0.90	0.24	21.08	43.30	9.66	21.16
MY2 (t ha^−1^)	0.09	0.99	0.22	19.51	41.69	10.70	18.83
CY (t ha^−1^)	2.95	16.88	0.34	26.04	44.43	5.73	31.49

a SDI = Stem diameter increment from 2012 to 2015; NOL = number of laterals; MY1 = First three years mean yield (2015–2017); MY2 = last three years mean yield (2018–2020); CY = cumulative yield from 2015 to 2020.

b δ^2^_g_ = genotypic variance; δ^2^_e_ = error variance; H^2^ = broad sense heritability; GCV = genotypic coefficient of variation; PCV = phenotypic coefficient of variation.

There was a significant effect of the environment on all the traits assessed and this resulted in low-to-moderate broad sense heritability estimates, varying from 0.06 for 2020 yield to 0.34 for CY. Broad sense heritability of the growth traits varied from 0.11 for SDI to 0.30 for number of laterals (Table 4). The genotypic coefficient of variation (GCV) was less than the phenotypic coefficient of variation (PCV) for all traits evaluated. Mean yield in 2016 had the highest GCV of 43.8% whereas SDI had the lowest (6.4%) GCV compared with the other traits. A similar trend was observed for the PCV where 2016 mean yield had the highest (105.2%) and span had the lowest of 13.2%.Genetic advance as a percentage of the trait mean (GAM) at a 5% selection intensity varied from 4.4% for stem diameter increment to a high of 37.5% for 2016 yield. Generally, the GAM for the vegetative growth traits were relatively low compared with the GAM for the yield traits (Table 4).

**Figure 1:**
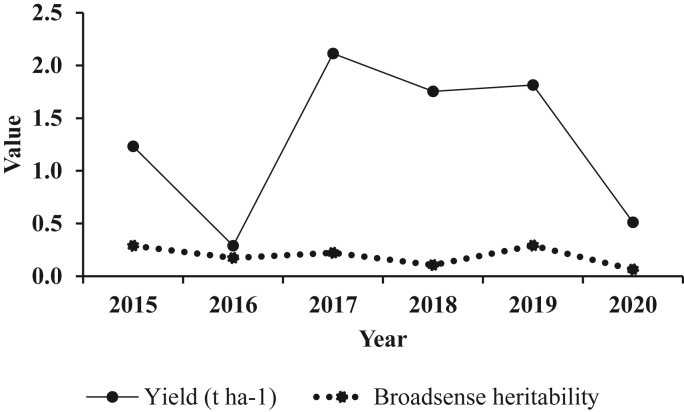
Pattern of average yield and heritability across years for 56 Robusta coffee clones evaluated.

The pattern of yield and broad sense heritability across years for the 56 Robusta coffee clones assessed are shown in Figure 1. In general, the mean yield was biennial with a peak in 2017. Also, generally the environmental variances and resultant broad sense heritability estimates for yield followed a biennial pattern where it was relatively high and low in high-yielding and low-yielding years, respectively (Figure 1). Although, broad sense heritability was biennial in nature similar to yield, it was fairly stable from 2015 to 2018 and peaked in 2019 followed by a decline in 2020 (Figure 1).

### 3.4 Genotypic and phenotypic correlations among traits

The phenotypic (*r*) and genetic (*r*_*g*_) correlations among the vegetative growth (stem diameter increments, SDI; span; number of laterals, NOL) and yield (2015–2020 mean yields; first 3-years average yield, MY1; last 3-years average yield, MY2 and cumulative yield, CY) traits measured are presented in Table 5. All phenotypic associations estimated were positive. On the other hand, a majority of the genetic associations were positive and significant. The few noteworthy exceptions were the association between stem diameter increments (SDI) and 2018 yield (*r*_*g*_ = -0.29, *p* < 0.05) and between 2016 yield and 2020 yield (*r*_*g*_ = -0.37, *p* < 0.01). In contrast, the phenotypic associations were significantly lower than their equivalent genotypic associations in most trait combinations (Table 5). Generally, the vegetative growth parameters had a stronger association between themselves rather than with the yield parameters. A noticeable variation was the association between the growth traits and 2020 yield (*r*_*g*_ = 0.98, *p* < 0.001 for SDI; *r*_*g*_ = 0.98, *p* < 0.001 for Span and *r*_*g*_ = 0.75, *p* < 0.001 for NOL). Among the growth parameters, the strongest association was found between number of laterals and span (*r*_*g*_ = 0.74, *p* < 0.001) (Table 5). The genetic associations between the yield traits were much larger than the associations between the vegetative growth traits, varying from (*r*_*g*_ = 0.67, *p* < 0.001) for (2015 yield vs MY2) to (*r*_*g*_ = 0.98, *p* < 0.001) for (2016 yield vs MY2; 2016 vs CY; 2017 yield vs MY1 and 2020 yield vs CY) (Table 5).

**Table 5:** Genetic (below diagonal) and phenotypic (above diagonal) correlations between vegetative growth and yield traits of 56 Robusta coffee clones evaluated.

Trait^a^	SDI	Span	NOL	Yield-15	Yield-16	Yield-17	Yield-18	Yield-19	Yield-20	MY1	MY2	CY
SDI (mm)	─	0.57∗∗∗	0.57∗∗∗	0.32∗	0.20ns	0.27ns	0.06ns	0.05ns	0.13ns	0.35∗∗	0.11ns	0.26ns
Span (m)	0.53∗∗∗	─	0.67∗∗∗	0.48∗∗∗	0.06ns	0.45∗∗∗	0.31∗	0.29∗	0.23ns	0.51∗∗∗	0.35∗∗	0.51∗∗∗
NOL	0.57∗∗∗	0.74∗∗∗	─	0.63∗∗∗	0.12ns	0.57∗∗∗	0.27∗	0.36∗∗	0.16ns	0.67∗∗∗	0.36∗∗	0.58∗∗∗
Yield-15 (t ha^−1^)	0.10ns	0.19ns	0.47∗∗∗	─	0.30∗	0.78∗∗∗	0.44∗∗∗	0.66∗∗∗	0.17ns	0.91∗∗∗	0.59∗∗∗	0.83∗∗∗
Yield-16 (t ha^−1^)	0.60∗∗∗	0.38∗∗	0.47∗∗∗	0.98∗∗∗	─	0.35∗∗	0.15ns	0.39∗∗	0.09ns	0.31∗	0.32∗	0.37∗∗
Yield-17 (t ha^−1^)	0.03ns	0.39∗∗	0.57∗∗∗	0.83∗∗∗	0.97∗∗∗	─	0.56∗∗∗	0.78∗∗∗	0.23ns	0.92∗∗∗	0.71∗∗∗	0.92∗∗∗
Yield-18 (t ha^−1^)	-0.29∗	0.24ns	0.17ns	0.51∗∗∗	0.98∗∗∗	0.70∗∗∗	─	0.55∗∗∗	0.31∗	0.50∗∗∗	0.83∗∗∗	0.72∗∗∗
Yield-19 (t ha^−1^)	0.05ns	0.29∗	0.34∗∗	0.81∗∗∗	0.98∗∗∗	0.92∗∗∗	0.83∗∗∗	─	0.21ns	0.67∗∗∗	0.88∗∗∗	0.85∗∗∗
Yield-20 (t ha^−1^)	0.98∗∗∗	0.98∗∗∗	0.75∗∗∗	0.98∗∗∗	-0.37∗∗	0.98∗∗∗	0.98∗∗∗	0.98∗∗∗	─	0.25ns	0.38∗∗	0.31∗
MY1 (t ha^−1^)	0.08ns	0.44∗∗∗	0.68∗∗∗	0.96∗∗∗	0.78∗∗∗	0.98∗∗∗	0.56∗∗∗	0.84∗∗∗	0.97∗∗∗	─	0.63∗∗∗	0.89∗∗∗
MY2 (t ha^−1^)	-0.05ns	0.40∗∗	0.33∗	0.67∗∗∗	0.98∗∗∗	0.78∗∗∗	0.93∗∗∗	0.95∗∗∗	0.96∗∗∗	0.72∗∗∗	─	0.86∗∗∗
CY (t ha^−1^)	-0.02ns	0.47∗∗∗	0.49∗∗∗	0.83∗∗∗	0.98∗∗∗	0.94∗∗∗	0.79∗∗∗	0.93∗∗∗	0.98∗∗∗	0.87∗∗∗	0.94∗∗∗	─

∗, ∗∗, ∗∗∗, ns denote significance at 0.05, 0.01, 0.001 and not significant, respectively.

a SDI = Stem diameter increment from 2012 to 2015; NOL = number of laterals; MY1 = First three years mean yield (2015–2017); MY2 = last three years mean yield (2018–2020); CY = cumulative yield from 2015 to 2020.

Genetic association showed that selection based on average yield in early years (i.e. first three years, MY1) would be as effective as selection based on cumulative yield (CY), adjudged by the relatively strong genetic correlation between MY1 and CY (*r*_*g*_ = 0.87, *p* < 0.001). Similarly, selection based on average early years’ yield (MY1) may be very effective as selection based on later years’ average yield (MY2) (*r*_*g*_ = 0.72, *p* < 0.001) (Table 5). Also, later years’ mean yield (MY2) was highly associated with cumulative yield (*r*_*g*_ = 0.94, *p* < 0.001). The genetic correlations further revealed that selection for plants with larger span could result in plants with a moderate increase in cumulative yield (*r*_*g*_ = 0.47, *p* < 0.001). Similarly, selection for coffee trees with increased number of laterals could result in trees with a moderate increase in cumulative yield (*r*_*g*_ = 0.49, *p* < 0.001) (Table 5). Both positive and negative covariance were observed among the growth and yield traits assessed in the present study, albeit a majority being positive. The least covariance (−0.014) was observed between 2016 yield and MY1 whereas the largest covariance (0.134) was observed between 2016 yield and 2020 yield (Table S2).

## 4 Discussion

In any crop enhancement program, knowing the genetic properties of target traits and the inter-relationships of these key agronomic traits is a pre-requisite in establishing a successful selection program. For this reason, our study sort to determine the agronomic performance and genetic variation among Robusta coffee clones in the coffee improvement program at the CRIG and decipher the genetic relationship among key traits of interest to guide future selection and hybridization efforts. Our findings build upon previous studies in coffee and other crops and will be relevant for informing future breeding strategies for Robusta coffee.

Generally, it is agreed that Robusta coffee is mainly a cross-pollinated species, hence a high level of genetic variability exists in this species. In corroboration with this assertion, our results revealed the presence of considerable variation among the clones for all the traits assessed. The existence and exploitation of such sufficient variability among the evaluated clones provides immense opportunity for development of better Robusta coffee varieties in the future. The significant variability observed for the study traits were in agreement with the finding of earlier researchers who reported significant genetic variability within Robusta (Akpertey et al., 2020; Cilas et al., 2006; Mistro et al., 2004; Petiard et al., 2004) and Arabica (Getachew et al., 2017; Lemi and Ashenafi, 2016; Olika et al., 2011; Cilas et al., 1998) coffee genotypes for important yield and growth traits. Similarly, Carvalho et al. (2008) found significant variation among coffee progenies developed through hybridization between commercial cultivars and Timor hybrid accessions which corroborates the findings of the present study. Our study further revealed a significant block effect for a majority of the traits assessed. This suggests that the study was conducted in a heterogeneous test environment and thus, the design and blocking adopted for the experiment was suitable. Also, 24 plants per clone in total were utilized in our study. This is suitable because clones are true-to-type and such a number has been shown to be adequate to determine average performance in clonally propagated tree crops (Cilas et al., 2010).

Cumulative yield is by far an important yield parameter to be considered in determining the overall yield potential of varieties of a perennial tree crop like coffee. From this study, the highest-yielding genotype was the standard clone, E139 with a cumulative yield of 15.5 t ha^−1^ over a six-year yield collection period. This suggested that significant efforts need to be made in the CRIG Robusta coffee breeding program through ortet selection of clones from hybridization efforts as well as introduction, evaluation and selection of high-yielding germplasm clones to identify newer higher-yielding clones to augment existing varieties. This is paramount considering the significance of high-yielding varieties in enhancing national coffee productivity. Regardless of the best clone being one of the standard clones, six new clones (H1001, H242, H207, H222, H760 and A129) were significantly better than the standard clone, E138 with cumulative yields ranging between 8.6 t ha^−1^ (H1001) to 10.6 t ha^−1^ (A129). These promising clones together with other genotypes may be good candidates for future multi location clonal evaluations.

Correlation analysis in plant breeding is important because it can help detect associations among target traits, identify new parental combinations in developing improved varieties and identify redundancy in trait measurements (Yan and Fregeau-Reid, 2008). Phenotypic correlation determines how traits vary together across phenotypes whereas genetic correlation on the other hand measures how same gene or genes that are closely linked control different traits (Silva et al., 2007). In population improvement or hybrid development, adequate understanding of genetic associations among traits is a key requirement in developing appropriate selection criteria to facilitate the crop improvement process. Given the cost of phenotyping large populations and how fast farmers want varieties developed and released for them, the trait associations found in this study could help facilitate the crop improvement process by making use of genetic associations for indirect selection. The genetic correlation analyses of the growth and yield traits showed that cumulative yield was positively and significantly influenced by span and number of laterals which was similar to the findings of Silvarolla et al. (1997) and Carvalho et al. (2010) whose study also revealed significant associations between growth traits and yield. This implied that coffee trees that had proper vegetative growth in early years of the crop can produce better yields in later years. Also, both traits (span and number of laterals) had relatively high heritability estimates in comparison with stem diameter increment. Priority should therefore, be given to span and number of laterals in selection due to their strong associations and relatively large magnitudes of direct effects on yield. Both traits (span and number of laterals) that were positively and significantly associated with cumulative yield can be employed in indirect selection for yield in Robusta coffee, thereby facilitating the efficiency in selection. When such selection is practiced early in a crop improvement program by disregarding inferior genotypes, the resources required in developing better varieties can be significantly reduced.

Van der Vossen (1985) posited that in any crop improvement program, selection efficiency for yield can be improved by determining and utilizing the information on the association between growth and yield traits. In agreement with this assertion, a strong genetic correlation (*r*_*g*_ = 0.87, *p* < 0.001) was found between the first three years’ mean yield (MY1) and cumulative yield (CY) as well as a strong genetic correlation (*r*_*g*_ = 0.72, *p* < 0.001) between the first three years’ mean yield (MY1) and the last three years’ mean yield (MY2). Variety development in a tree crop like coffee can take several years to achieve, given how long it takes to measure yield effectively. Such a strong genetic correlation between early years’ mean yield and cumulative yield suggests that selection for yield could be practiced early in a breeding program to achieve similar gains as when selections were performed on later years’ mean yield or cumulative yield. Stem diameter increment was poorly associated with cumulative yield from the present study which is in disagreement with findings of Akpertey et al. (2020) who found significant relationship between stem diameter and cumulative yield among Robusta coffee hybrids. This disparity could be due to the differences in genetic structure of the genotypes assessed in both studies. Generally, both number of laterals and span correlated strongly with cumulative yield than with yearly yields, with the exception of 2020 yields. This suggests that there may have been less inter tree competition among the test genotypes in the present study as was shown by Leroy et al. (1994). These results also indicated that direct selection of clones with greater number of laterals or larger span could result in an indirect increase in yield in Robusta coffee. Thus, vegetative growth traits like number of laterals and span can be the focus in breeding programs to indirectly select for varieties with higher yield.

Despite the importance of genetic correlations in any plant breeding program, there are potential trade-offs for genetic gains throughout breeding that may be targeted at developing improved varieties. A strong genetic correlation between a set of traits may result in a possible significant genetic gain in a trait that is indirectly selected for. The reverse is the case when the genetic correlations between the traits of interest is weak. This is particularly important given the usually complex nature of the covariance matrix among target traits (Sedlacek et al., 2016) where there could be a strong genetic correlation between two traits but a weak correlation with a third indirect target trait. Trade-off between traits is usually the result of an unfavorable correlation between them (Garland, 2014) such that attempts to improve the third indirect target trait may require a compromise on genetic gain for the second indirect target trait or even the main trait. Also, the traits assessed in the present study related to adaptation are usually polygenic in nature (Cortés et al., 2018) and this may limit the efficiency of selection and hence, genetic gain. Additionally, in most breeding programs, genotypes may be evaluated across several sites or over many years resulting in the interaction between genotype and environment or years in trait expression. A significant genotype × year interaction for target traits as a result of changes in genotype performance over years may limit the efficiency of selection. However, in such situations, selection based on cumulative trait performance over time may result in better selection efficiency and genetic gains for important traits. For the present study, the trait covariance matrix revealed that there is enough heritable uncorrelated variation among the target traits upon which efficient selection could be undertaken that could result in significant genetic gains.

The variance due to the environment was larger than the variance due to genetic factors for all the traits assessed in the present study, indicating the predominance of environmental variance to the detriment of genetic factors. This finding suggests that predictability of family performance would be difficult as families with good trait measurement in one year may have varied response in different seasons. Nonetheless, increased sampling of the experimental area through increased number of trees per replication may be essential to increase the accuracy of observed genotypic differences and reduce the variance due to error in future studies. The findings of our study especially regarding heritability estimates demonstrated that there may be heritable variation upon which selection could act due to changing environmental factors. However, a limitation in the present study is the lack of evaluating the genotypes in contrasting environments as well as the lack of explicit estimates of selection coefficients from fitness vs. trait values regressions that give credence and more impetus to the potential of making significant gains from selection via conventional clonal selection (Sedlacek et al., 2015). Since these estimates (i.e. fitness vs. trait values regressions) were not gathered in the present work and the genotypes were evaluated in only one location, caution must be undertaken in concluding that there is significant potential to improve the tested genotypes via conventional clonal selection. Future evaluation of these genotypes in contrasting environments would be worthwhile to better estimate and conclude on the level of attainable genetic gain when conventional clonal selection is practiced.

To develop improved varieties, it is imperative that coffee breeders have target traits that have high heritability, positively correlate with other traits and exist in populations with significant genetic variations to allow for better trait improvement. Broad sense heritability is the amount of phenotypic variation that is due to total (additive and non-additive) genetic effects and is vital in clonally propagated crops like Robusta coffee where clonal propagation of elite individual genotypes can exploit this source of variation (Silva et al., 2007). Only broad sense heritability which does not distinguish dominance and epistatic genetic variance from the additive component was possible to estimate in the present study due to the clonality of the genotypes. As proposed by Verma and Agarwal (1982), the estimates of heritability for a majority of the traits in our study were moderate (0.20–0.50). This was very well in line with the findings of other authors who worked on Robusta coffee (Cilas et al., 2006; Anim-Kwapong and Anim-Kwapong, 2012; Akpertey et al., 2020). Among the growth and yield traits, number of laterals (0.30) and cumulative yield (0.34), respectively had the highest heritability estimates. In comparison with yearly yields, the effect of the environment on the expression of cumulative yield was relatively low as evidenced by the relatively low (5.73) ratio of variance due to environmental factors to variance due to genetic factors. This suggests that selection for yield in a high-yielding year may vary from selection in a low-yielding year. Hence, selection for yield in Robusta coffee should be based on the cumulative performance of clones or average of yield over a number of years and not on yearly yield performance. According to Nair et al. (2012), a high broad sense heritability indicates that, a trait is least influenced by environmental factors, and the selection for improvement of such a trait would be valuable, because broad sense heritability is based on total genetic variance which comprises both additive; and dominance and epistatic variances.

There was marked variation in broad sense heritability for the five yearly harvests for yield from 2015 through 2020. The variation followed a biennial pattern where a low estimate of heritability in one year was followed by a high estimate of heritability for yield in the subsequent year. The different estimates of heritability for the six yearly harvests and cumulative yield are probably a result of differences in gene expression during plant growth and development, differences in size, vigor and early seedling growth following field transplanting as well as prevailing conditions of the environment in the year harvesting is undertaken.

Estimates of genetic parameters permit the breeder to better understand the nature of the gene action involved in inheritance of traits, allow for better assessment of the progress expected with selection, as well as help to define the best selection strategies to be employed in any breeding program (Oliveira et al., 2008). Estimation of genotypic (GCV) and phenotypic coefficient of variations (PCV) from the present study revealed that the coffee clones expressed varying levels of variations in growth and yield traits. The GCV estimates for the growth traits were in general lower than that of the yield traits, suggesting that variability in the growth traits was more due to non-genetic factors. The magnitude of the variations between genotypic and phenotypic coefficients of variation determines degree to which the environment influences the phenotypic manifestation of any character. Large differences reflect significant effect of the environment, whereas small differences reveal significant effect of genetic factors (Akinwale et al., 2011). For all the traits evaluated, the estimates of PCV were higher than those estimated for the GCV, suggesting that a combination of both environmental and genotypic effects influenced the expression of these traits. This corroborates the findings of Akpertey et al. (2020) and suggests that there may be variability in response of clones over several years, making it difficult to maintain predictability. To guide breeding efforts, Johnson et al. (1955) proposed a GAM categorization as low (0–10%), medium (10–20%) and high (>20%). Based on this categorization, when the top 5% of clones were selected, two (stem diameter increments and span) of the three growth traits had a low GAM whereas number of laterals had a medium GAM of 13.95%. Of the yield traits, low (8.73%) GAM was recorded for 2020 yield whereas medium GAM was observed for 2018 yield (10.37%) and mean yield for the last three years of yield recording (MY2: 18.83%). Yield in 2015, 2016, 2017, 2019, mean yield for the first three years (MY1) and cumulative yield had high GAM. The high GAM observed for most of the yield traits suggested additive and non-additive genes also contributed to trait expression in addition to environmental effects (Abate et al., 2015).

Generally, the PCV for the yield traits were much higher than GCV, leading to relatively higher GAM for the same traits. This suggests that the traits with high GAM are controlled more by additive genes as proposed by Panse (1967), therefore, selection for crop improvement based on these traits would be quite effective. The high GAM observed for yield traits in this study are in agreement with the findings of Olika et al. (2011) and Bayetta et al. (2007). The GAM for the growth traits however, were low-to-moderate, in agreement with the findings of Malau and Pandiagan (2018) who also reported low-to-moderate GAM for many plant vigor traits. Similarly, this corroborates the findings of Akpertey et al. (2020) who working on Robusta coffee hybrids reported low-to-moderate GAM for growth traits and comparatively high GAM for yield traits at 5% selection intensity. The relatively high estimates of heritability observed for number of laterals and cumulative yield combined with the relatively high GAM estimates would lead to significant enhancement of both traits in a breeding program through selection because the use of heritability in combination with genetic advance has been reported to be critical for the effective enhancement of target traits in a breeding population (Yigzaw, 2005).

In conclusion, the present study has revealed the presence of significant genetic variability among the 56 Robusta coffee clones for key agronomic traits that could be exploited in future coffee improvement programs. For an effective selection, the combined use of heritability and genetic advance is vital for assessing the attainable level of genetic improvement from selection of target traits. The highest broad sense heritability for the growth and yield traits were observed for number of laterals and cumulative yield, respectively. This coupled with the relatively high GAM for both traits suggests that direct selection for number of laterals and cumulative yield would result in an increase in the frequency of favorable alleles for both parameters in the population that would lead to significant gains from selection in our breeding program. The significant genetic association found between early years’ yield (MY1) and cumulative yield in our study suggests that selection in early years could be as effective as selection carried out in later years, thereby facilitating the crop improvement process.

## 5 Prospects for future studies

One major finding of the present study was the significant clone by year interaction when a pooled ANOVA was conducted. Also, there were differences in yield across years depicting a biennial pattern which has significant implications for improvement in selection efficiency in a crop improvement program. Considering that the clones were tested in a single location over several years in the present study, invites strongly considering whether environmental/year plasticity may be further studied in our crop improvement program and other similar programs. Better modeling of genotype by environment/year plasticity in Robusta coffee would require testing all evaluated clones or similar ones in multi environments over several years which would allow for improving selection efficiency and gains from selection for important traits in any crop improvement program (Cortés et al., 2020). This is particularly vital since extensive phenotypic changes (Hallingback et al., 2019) to climate variabilities is apparent in trees, and strongly justifies the need to identify resilient genotypes through a lengthy gestation period. Another necessary vital step to improve selection efficiency would involve exploring the underlying genomic basis of the heritable variation observed in the present study. One approach worth pursuing in investigating the observed genetic variation to side step the difficulties of genotype by environment/year plasticity in tree crop improvement is predictive genomics, that incorporates the use of molecular markers, with the prospect of increasing selection accuracy within commercial parameters as well as shorten intervals within generations for selection (Grattapaglia et al., 2018; Arenas et al., 2021), assist the identification of interesting variants from germplasm (Migicovsky and Myles, 2017), and reveal the genomic prospect of adaptation to variable climates (Lind et al., 2018). Also, machine learning techniques will brace genetic predictions aimed at several traits in multi-location experiments that aim at disentangling the additive genetic variance from the variance resulting from genotype by environment factors that would result in an improvement in efficiency of selection and gains from selection (Cortés et al., 2020) in crop improvement. All these efforts enumerated above will eventually apprise germplasm conservation and development to improve selection efficiency as well as genetic advance of important agronomic parameters of Robusta coffee which would result in increased productivity.

## Declarations

### Author contribution statement

Abraham Akpertey, Esther Anim-Kwapong: Conceived and designed the experiments; Performed the experiments; Analyzed and interpreted the data; Wrote the paper.

Paul Kwasi Krah Adu-Gyamfi, Atta Ofori: Performed the experiments; Analyzed and interpreted the data; Wrote the paper.

### Funding statement

This research did not receive any specific grant from funding agencies in the public, commercial, or not-for-profit sectors.

### Data availability statement

Data included in article/supplementary material/referenced in article.

### Declaration of interest’s statement

The authors declare no conflict of interest.

### Additional information

No additional information is available for this paper.
